# Limited impact of *Cntn4* mutation on autism-related traits in developing and adult C57BL/6J mice

**DOI:** 10.1186/s11689-016-9140-2

**Published:** 2016-03-02

**Authors:** Remco T. Molenhuis, Hilgo Bruining, Esther Remmelink, Leonie de Visser, Maarten Loos, J. Peter H. Burbach, Martien J. H. Kas

**Affiliations:** Department of Translational Neuroscience, Brain Center Rudolf Magnus, University Medical Center Utrecht, Utrecht, The Netherlands; Department of Psychiatry, Brain Center Rudolf Magnus, University Medical Center Utrecht, Utrecht, The Netherlands; Sylics (Synaptologics BV), Amsterdam, The Netherlands; Department of Molecular and Cellular Neurobiology, Center for Neurogenomics and Cognitive Research, Neuroscience Campus Amsterdam, VU University, Amsterdam, The Netherlands; Department of Functional Genomics, Center for Neurogenomics and Cognitive Research, Neuroscience Campus Amsterdam, VU University, Amsterdam, The Netherlands

**Keywords:** *CNTN4*, Autism spectrum disorder, 3p deletion syndrome, Developmental trajectories, Mouse model, Behavior, Reversal learning, Negative findings, Hyperreactivity, Schizophrenia

## Abstract

**Background:**

Mouse models offer an essential tool to unravel the impact of genetic mutations on autism-related phenotypes. The behavioral impact of some important candidate gene models for autism spectrum disorder (ASD) has not yet been studied, and existing characterizations mostly describe behavioral phenotypes at adult ages, disregarding the developmental nature of the disorder. In this context, the behavioral influence of *CNTN4*, one of the strongest suggested ASD candidate genes, is unknown. Here, we used our recently established developmental test battery to characterize the consequences of disruption of *contactin 4* (*Cntn4*) on neurological, sensory, cognitive, and behavioral phenotypes across different developmental stages.

**Methods:**

C57BL/6J mice with heterozygous and homozygous disruption of *Cntn4* were studied through an extensive, partially longitudinal, test battery at various developmental stages, including various paradigms testing social and restricted repetitive behaviors.

**Results:**

Developmental neurological and cognitive screenings revealed no significant differences between genotypes, and ASD-related behavioral domains were also unchanged in *Cntn4*-deficient versus wild-type mice. The impact of *Cntn4*-deficiency was found to be limited to increased startle responsiveness following auditory stimuli of different high amplitudes in heterozygous and homozygous *Cntn4*-deficient mice and enhanced acquisition in a spatial learning task in homozygous mice.

**Conclusions:**

Disruption of *Cntn4* in the C57BL/6J background does not affect specific autism-related phenotypes in developing or adult mice but causes subtle non-disorder specific changes in sensory behavioral responses and cognitive performance.

**Electronic supplementary material:**

The online version of this article (doi:10.1186/s11689-016-9140-2) contains supplementary material, which is available to authorized users.

## Background

Autism spectrum disorder (ASD) is a behaviorally defined developmental disorder with a strong genetic component [1, 2]. The identification of genetic risk factors such as common genetic variants, rare inherited and *de novo* mutations have lead to the implication of hundreds of different genes [3, 4]. These findings illustrate the complexity and heterogeneity of the genetic architecture of ASD.

Subsequently, mouse models are being used to unravel the functional impact of implicated genes on ASD phenotypes in a controlled genetic and environmental background. However, knowledge of the impact on cognitive and behavioral development of the majority of these genes is missing or incomplete [5], and most behavioral characterizations of animal models are limited to adult phenotypes disregarding the developmental nature of ASD [6].

Here, we characterize the impact of *contactin 4* (*Cntn4*) null mutation on behavioral development, using our recently developed longitudinal test battery for mice that tests a wide array of neurological, cognitive, and behavioral parameters across development starting from 3 weeks of age [6].

CNTN4 is an axonal glycoprotein belonging to the contactin family, a six-member subgroup of the immunoglobulin superfamily of cell adhesion molecules [7]. CNTN4 is known to act as an axon guidance molecule in the establishment of olfactory neural circuitry during neural development [8] and promotes target-specific axon arborization of a subset of retinal ganglion cells onto the nucleus of the optic tract [9]. Knowledge of the neurobiological functions of CNTN4 in normal and abnormal development of brain systems are far from complete.

The *CNTN4* gene has been implicated in ASD due to its presence in the genetic locus of the 3p-deletion syndrome, a mental retardation syndrome [10]. Subsequently, evidence for a role of CNTN4 has been accumulated [11–14] but has also been questioned [15, 16]. Three cases carrying a copy number variant (CNV) in the *CNTN4* gene were reported by the Autism Genome Project Consortium (AGP) [12]. Deletions and duplications in the *CNTN4* gene or its promoter region were found in 10 families of the Autism Genetic Resource Exchange (AGRE) collection [13]. In the Autism Case-Control cohort (ACC), deletion in the promoter region of *CNTN4* was found in three cases but not in controls [13]. Association of *CNTN4* with developmental disorders such as ASD further seems supported by the protein’s neurobiological functions [2, 7].

Follwoing these observations, we performed a careful longitudinal functional characterization of homozygous and heterozygous disruptions of *Cntn4* to resolve the impact of this gene on behavioral and cognitive development.

## Methods

### Generation and breeding of *Cntn4* mice

*Cntn4*-deficient mice were kindly provided by Dr. Yoshihiro Yoshihara (RIKEN, Japan) [8]. These mice were generated using a standard gene-targeting method as previously described. A targeting vector was designated to mutate the translation start codon (ATG) in the exon 2 of the *Cntn4* gene into a stop codon (TAG) and introduce a pgk-neo selection marker. Consequently, these mice were backcrossed with C57BL/6 mice more than nine times. Upon arrival in the University Medical Center Utrecht, the mice were re-derived, followed by heterozygous breeding for the use in our experiments.

All animals were born and weaned at the University Medical Center Utrecht. Average nest size of Cntn4 litters was 7.2, and the litters larger than 10 animals per litter were culled back to average 7. The minimum litter size used for behavioral experiments was four animals per litter. Detailed information on the genotyping of *Cntn4* mice is provided in Additional file 1.

Given the extensive number of tests, the mice were spread over four different batches. Table 1 provides the order of behavioral testing and the number of animals per genotype per batch. Phenotypic assessments in batches 1, 2, and 3 were performed at the University Medical Center Utrecht. Batch 4 was transported and tested at Sylics (Synaptologics BV, Amsterdam, The Netherlands).

**Table 1:** Overview of the behavioral tests per batch

Age	Task	Batch	Subjects (*n*)
3 weeks	Juvenile social interaction	Batch 1 + 2	6–7 genotype-matched pairs per genotype
4 weeks	Extended SHIRPA screen	Batch 1 + 2	19–26 per genotype
6 weeks	Extended SHIRPA screen	Batch 1 + 2	19–26 per genotype
8 weeks	Extended SHIRPA screen	Batch 1 + 2	19–26 per genotype
Adult	Extended SHIRPA screen	Batch 1	10–15 per genotype
	Open field	Batch 1	10–15 per genotype
	Elevated plus maze	Batch 1	10–15 per genotype
	Social discrimination	Batch 1	10–15 per genotype
	Buried food test	Batch 2	9–11 per genotype
	Set shifting-reversal task	Batch 2	9–11 per genotype
	Social approach in 3-chamber	Batch 3	12 per genotype
	Novel object exploration task	Batch 3	12 per genotype
	Barnes maze-reversal task	Batch 4	16 per genotype
	Pre-pulse inhibition	Batch 4	16 per genotype

Animals in batch 1 + 2 were weaned at post-natal day 21. Batch 3 + 4 animals were weaned at post-natal day 28

All experiments were approved by the ethical committee for animal experimentation of the University Medical Center Utrecht and Free University Amsterdam and performed according to the institutional guidelines that are in full compliance with the European Council Directive (86/609/EEC).

### Developmental neurological and behavioral screening

*Cntn4*^*−/−*^, *Cntn4*^*+/−*^, and wild-type male littermates were subjected to our previously described longitudinal screening strategy (extended SHIRPA battery) testing an array of neurological, behavioral, and cognitive parameters at 4, 6, 8, and 11 weeks of age [6, 17]. The longitudinal test battery includes the assessment of autism-related traits such as motor stereotypies (e.g., self-grooming) and sensorimotor coordination (e.g., latency to fall from the rotarod). Detailed information on behavioral testing is provided in Additional file 1.

### Screening of social behaviors and restricted repetitive behaviors

Abnormalities in social interaction behaviors were assessed in the juvenile social interaction test (3 weeks of age) [18], followed by a three-chamber social approach [18], and a 2-day social discrimination paradigm in adult age animals [19]. Stereotypic movements, restricted interests, and repetitive patterns of behavior were analyzed in the novel object investigation task during exposure to four novel toys [20]. Cognitive flexibility was assessed by multi-trial associative learning in an extensive set-shifting paradigm [6], as well as a Barnes maze spatial learning task including reversal [21]. Acoustic startle response and sensorimotor gating were assessed in the pre-pulse inhibition (PPI) test [19]. Anxiety-related behaviors were tested in the elevated plus maze and open field [6]. Statistical analyses are described in the Additional file 1.

## Results

### eSHIRPA assays

**Fig. 1:**
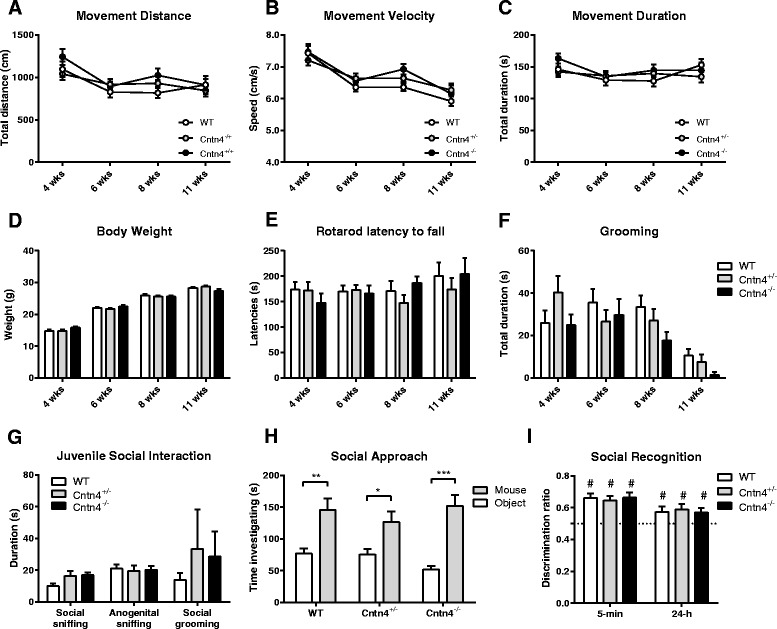
Developmental neurological and behavioral screen and analysis of social behaviors in *Cntn4-*deficient mice. **a** Distance moved (rmANOVA genotype, *F*_(2,63)_ = 0.760, *p* = .472), **b** movement velocity (rmANOVA genotype, *F*_(2,63)_ = 1.256, *p* = .292), **c** movement duration (rmANOVA genotype, *F*_(2,63)_ = 0.342, *p* = .479), **d** body weight (rmANOVA genotype, *F*_(2,63)_ = 0.588, *p* = .558), **e** latency to fall of the accelerating rotarod (rmANOVA genotype, *F*_(2,64)_ = 0.110, *p* = .896), and **f** time spent self-grooming (rmANOVA genotype, *F*_(2,64)_ = 1.038, *p* = .360) at pre-adolescence (4 weeks), adolescence (6 weeks), early adulthood (8 weeks), and adulthood (10 weeks) (*n* = 19–26 per genotype) during the eSHIRPA test. **g** Social sniffing (owANOVA, *F*_(2,16)_ = 2.926, *p* = .083), anogenital sniffing (owANOVA, *F*_(2,16)_ = 0.055, *p* = .946), and social grooming (owANOVA, *F*_(2,16)_ = 0.334, *p* = .721) during the juvenile social interaction test in genotype-matched mice at post-natal day 21 (*n* = 6–7 pairs of genotype-matched interacting animals per genotype). **h** Social exploration (owANOVA between genotypes, *F*_(2,32)_ = 0.599, *p* = .556) as a function of exploration of the cage with the novel mouse versus the empty cage during the three-chamber task (*n* = 12 per genotype). **i** Social exploration during the social discrimination test following a 5-min inter-trial interval (owANOVA between genotypes *F*_(2,36)_ = 0.138, *p* = .872) and a 24-h inter-trial interval (owANOVA between genotypes *F*_(2,36)_ = 0.096, *p* = .909), with exploration of the novel mouse as fraction of the total duration of social exploration (*n* = 10–15 per genotype). Data are presented as means ± SEM. *p < 0.05; **p < 0.01; ***p < 0.001; #*p* < 0.05

The eSHIRPA (extended SmithKline Beecham, Harwell, Imperial College and Royal London Hospital phenotype assessment) screen did not show differences between the *Cntn4*^*+/−*^ mice, *Cntn4*^*−/−*^ mice, and wild-type controls at 4, 6, 8, or 10 weeks of age in general health, body weight, and neurological reflexes nor in the development of various locomotor parameters including total distance moved, movement velocity, and movement duration (Figs. 1a–d, Table 2). Moreover, we found no developmental differences in the amount of self-grooming or sensorimotor coordination on the rotarod (Figs. 1e–f).

**Table 2:** Physical and neurological features during the different phases of the extended SHIRPA primary screen.

Age (weeks)	4				6				8				Adult			
Test	WT	+/−	−/−	Sig.	WT	+/−	−/−	Sig.	WT	+/−	−/−	Sig.	WT	+/−	−/−	Sig.
Subjects (*n*)	19	26	22		19	26	22		19	26	22		13	15	10	
Body position (active)	95	96	100	0.22	100	100	100	1.00	100	96	100	0.46	100	100	100	1.00
Body position (inactive)	0	4	0		0	0	0		0	4	0		0	0	0	
Body position (excessive activity)	5	0	0		0	0	0		0	0	0		0	0	0	
Tremor (absent)	100	100	100	1.00	100	100	100	1.00	100	100	100	1.00	100	100	100	1.00
Palpebral closure (eyes open)	100	100	100	1.00	100	100	100	1.00	100	100	100	1.00	100	100	100	1.00
Coat appearance tidy and groomed	100	100	100	1.00	100	100	100	1.00	100	100	100	1.00	100	100	100	1.00
Whiskers (present)	100	100	100	1.00	100	100	100	1.00	100	100	100	1.00	100	100	100	1.00
Lacrimation (absent)	100	96	100	0.45	100	100	100	1.00	100	100	100	1.00	100	100	100	1.00
Defecation (quantity)	1.9	1.6	2.0	0.47	3.1	2.5	1.9	0.06	2.8	2.3	2.4	0.61	3.8	3.7	3.2	0.70
± SEM	0.3	0.3	0.3		0.3	0.3	0.3		0.4	0.3	0.4		0.7	0.7	0.9	
Transfer arousal (brief freeze)	11	15	14	0.70	11	19	9	0.71	11	8	0	0.16	8	0	10	0.50
Transfer arousal (immediate movement)	89	85	86		89	81	91		89	92	100		92	100	90	
Gait (fluid)	100	100	100	1.00	100	100	100	1.00	100	100	100	1.00	100	100	100	1.00
Tail elevation (horizontal extension)	100	100	100	1.00	95	100	100	0.28	100	100	100	1.00	100	100	100	1.00
Tail elevation (straub tail)	0	0	0		5	0	0		0	0	0		0	0	0	
Startle response (preyer reflex)	100	100	100	1.00	100	100	100	1.00	100	100	100	1.00	100	100	100	1.00
Touch escape (flight prior to touch)	79	73	77	0.88	89	85	73	0.35	63	88	68	0.05	85	87	100	0.45
Touch escape (response to touch)	21	27	23		11	15	27		37	12	32		15	13	0	
Positional passivity (struggles)	100	100	100	1.00	100	100	100	1.00	100	100	100	1.00	100	100	100	1.00
Skin color (pink)	95	100	100	0.28	100	100	100	1.00	100	100	100	1.00	100	100	100	1.00
Skin color (blanched)	5	0	0		0	0	0		0	0	0		0	0	0	
Trunk curl (absent)	100	100	100	1.00	100	100	100	1.00	100	100	100	1.00	100	100	100	1.00
Limb grasping (absent)	100	92	100	0.20	100	100	100	1.00	100	100	100	1.00	100	100	100	1.00
Pinna reflex (present)	100	100	100	1.00	100	100	100	1.00	100	100	100	1.00	100	100	100	1.00
Corneal reflex (present)	100	100	100	1.00	100	100	100	1.00	100	100	100	1.00	100	100	100	1.00
Contact righting reflex (present)	100	100	100	1.00	100	100	100	1.00	100	100	100	1.00	100	100	100	1.00
Evidence of biting (none)	79	77	77	0.99	84	88	100	0.18	100	100	100	1.00	100	100	100	1.00
Grip (OK)	100	96	100	0.36	95	96	100	0.59	100	100	100	1.00	100	100	90	0.24
Full puberty	89	81	100	0.30	100	100	100	1.00	100	100	100	1.00	100	100	100	1.00

*Cntn4* ^+/−^, *Cntn4* ^−/−^, and wild-type (WT) control mice were screened in the Perspex jar for body position (active, inactive, or excessively active), tremor (present or not), palpebral closure (eyes open or not), coat appearance (well groomed or irregularities like piloerection), whiskers (intact or trimmed), and lacrimation (present or not). In the arena, the mice were screened for transfer arousal (freezing or immediate movement), gait (fluid or not), tail elevation (dragging, horizontal, or straub tail), startle response (preyer reflex, no response, or additional response), and touch escape (response to touch or flight prior to touch while finger approaches). Mice were transferred out of the arena to observe positional passivity (struggling by different types of handling), skin color (color of plantar surface of forelimbs), trunk curl (forward curling with head to abdomen), limb grasping (clasping of rear limbs), pinna reflex (presence of ear retraction), corneal reflex (presence of eyeblink), contact righting reflex, evidence of biting, grip (grasping of grid), vocalization, and puberty (presence of sex organs). *Sig. *represents statistical significance of between-genotype differences. Data are presented as a percentage of the total number of animals per genotypes, except for defecation (count of the fecal boli)

### Social interaction behavior

The juvenile social interaction test revealed no differences in the amount of social sniffing, anogenital sniffing, or social grooming (Fig. 1g) between the *Cntn4*^*+/−*^, *Cntn4*^*−/−*^, and wild-type control mice. In the adult three-chamber test, all genotypes showed a clear preference for exploration of a mouse over an object, and no genotype differences in the amount of social exploration were observed (Fig. 1h). Furthermore, genotypes were equally capable to distinguish between a familiar mouse and a novel mouse in a social recognition paradigm, both at 5 min and 24 h after initial exposure (Fig. 1i).

### Restricted and repetitive behaviors

**Fig. 2:**
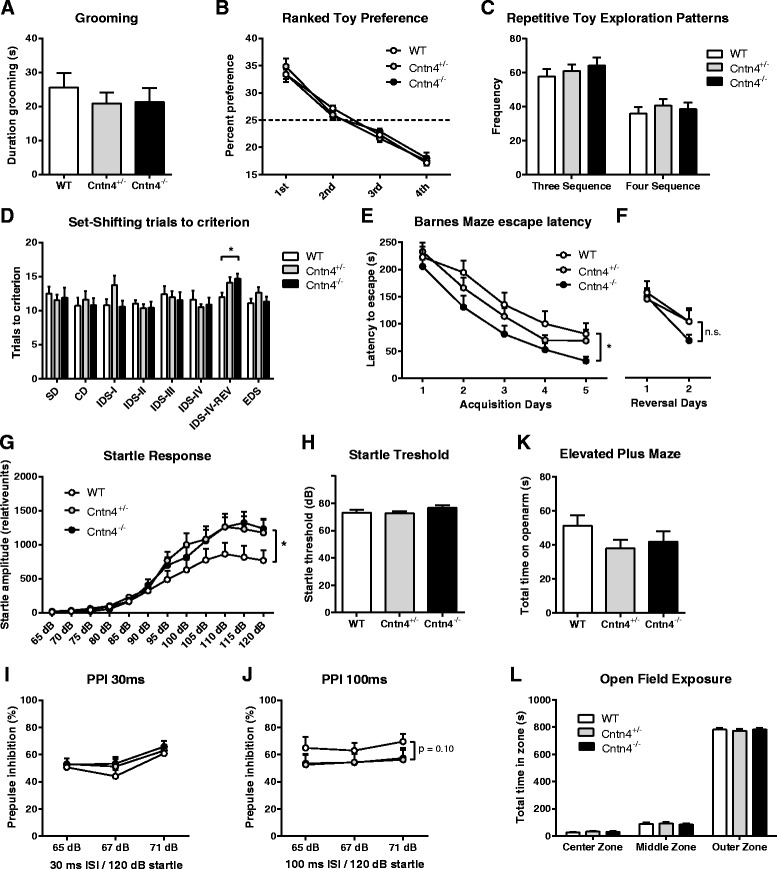
Restricted repetitive behaviors and sensory-sensitivity screening of adult *Cntn4* mice. Restricted and repetitive behavior in the novel object investigation task. **a** Stereotypic movements as total time spent grooming (owANOVA, *F*_(2,33)_ = 0.431, *p* = .653). **b** Restricted interest as frequency-based percentage preference of exploration of each of the four novel toys (1st preference owANOVA, *F*_(2,33)_ = 0.446, *p* = .644; 2nd preference owANOVA, *F*_(2,33)_ = 1.569, *p* = .223; 3rd preference owANOVA, *F*_(2,33)_ = 1.208, *p* = .312; 4th preference owANOVA, *F*_(2,33)_ = 0.236, *p* = .791). **c** Repetitive toy exploration patterns based on repetitive sequences of three elements (owANOVA, *F*_(2,33)_ = 0.760, *p* = .476) and four elements (owANOVA, *F*_(2,33)_ = 0.227, *p* = .798) (*n* = 12 per genotype). **d** Reversal learning during the set-shifting reversal-learning task. *X*-axis represents the different sub-tasks. *Y*-axis represents the total number of trials that were needed to reach the criterion of 8 correct digs in 10 consecutive trials (*n* = 9–11 per genotype). **e–f** Spatial learning and reversal learning during the Barnes maze paradigm. *Y*-axis represents the daily mean of latency to find the escape hole during **e** the acquisition phase (rmANOVA genotype, *F*_(2,44)_ = 4.151, *p* = .022) and **f** the reversal-learning phase (rmANOVA genotype, *F*_(2,43)_ = 0.830, *p* = .830) after replacing the escape to the other side of the maze (*n* = 16 per genotype). Startle and PPI results in *Cntn4* mice, with **g** startle magnitude as function of startle stimulus in all genotypes (MANOVA, *F*_(24,70)_ = 1.984, *p* = 0.014), **h** startle threshold (owANOVA, *F*_(2,45)_ = 1.542, *p* = 0.225), **i–j** pre-pulse inhibition tested with different pre-pulse intensities with inter-stimulus interval (ISI) at 30 ms (two-way ANOVA, *F*_(2,135)_ = 2.376, *p* = 0.096) and at 100 ms (two-way ANOVA, *F*_(2,135)_ = 1.1927, *p* = 0.306; *n* = 16 per genotype). Anxiety behavior during the elevated plus maze test and open-field test measured as **k** elevated plus maze anxiety and as total time spent on the open arms (owANOVA, *F*_(2,36)_ = 1.450, *p* = 0.248), **l** total time spent in the center (owANOVA, *F*_(2,36)_ = 0.165, *p* = 0.848), middle (owANOVA, *F*_(2,36)_ = 0.413, *p* = 0.665), and outer zones (owANOVA, *F*_(2,36)_ = 0.125, *p* = 0.883) of the open field (*n* = 10–15 per genotype). Data are presented as means ± SEM; *p < 0.05. *SD* simple discrimination, *CD* compound discrimination, *IDS I–IV* intra-dimensional shift I–IV, *IDS-reversal* reversal of intra-dimensional shift IV (owANOVA, *F*_(2,27)_ = 3.487, *p* = .045; Dunnett’s *t*, WT vs HET *p* = 0.037, WT vs KO *p* = 0.092), *EDS* extra-dimensional shift (owANOVA, *F*_(2,27)_ = 1.416, *p* = .260)

There were no differences in the grooming behavior between the *Cntn4*^*+/−*^, *Cntn4*^*−/−*^, and wild-type control mice at adult age, in line with the amount of grooming observed in the longitudinal eSHIRPA screening (Fig. 2a). We also found no genotype differences in restricted interest or in repetitive patterns of behavior in the novel object investigation task during the exploration of the four novel toys (Fig. 2b, c).

We additionally analyzed cognitive flexibility through the assessment of reversal learning in a set-shifting task. Prior to this task, we ascertained intact olfactory capacities, as we found no genotype differences in the latency to find a buried piece of food. In the set-shifting task, all genotypes were equally able to associate a food reward with a specific digging material or odor, as was evident through the performance of simple, compound discrimination and intra- and extra-dimensional shifts (Fig. 2d).

The reversal-learning phase of this test yielded an inconclusive result, as the genotype effect that we observed on reversal learning was only significant (ANOVA *p* = 0.04) in one of the two outcome measures (i.e., the number of trials to reach the criterion but not in errors to reach criterion). Moreover, a shift cost in wild-type mice was observed in errors to reach criterion, although not in the number of trials to reach criterion. Given this inconclusive result, we tested reversal-learning performance in a different paradigm. In this Barnes maze reversal-learning paradigm, we confirmed that *Cntn4 *does not affect reversal learning, as all genotypes needed equal amount of time as well as distance before reaching the re-located escape hole (Fig. 2f). In contrast to the set-shifting test, we observed a shift cost that was observed in all genotypes in the reversal phase of the Barnes maze test (Fig. 2e, f).

### Responses to sensory stimuli and anxiety-related behaviors

The startle response was consistently increased in the *Cntn4*^*+/−*^ and *Cntn4*^*−/−*^ mice at different high amplitudes, although the startle threshold was not significantly different between genotypes (Figs. 2g, h). No significant effects were found on pre-pulse inhibition at both inter-stimulus intervals of 30 and 100 ms (Figs. 2i, j).

The increased startle response to auditory stimuli of the different high amplitudes seemed not to result from increased anxiety levels, as *Cntn4*^*+/−*^, *Cntn4*^*−/−*^ mice and wild-type controls did not differ in their elevated plus maze (Fig. 2k) and open-field exploratory behaviors (Figs. 2l).

## Discussion

We present a comprehensive assessment of the impact of the ASD candidate gene *CNTN4* on a variety of neurological, behavioral, and cognitive aspects of development. We found no effect of *Cntn4* deficiency on ASD-related behavioral mouse paradigms such as the juvenile social interaction test, the three-chamber test, grooming behavior, and sensorimotor coordination. We also did not observe developmental neurological, behavioral, or cognitive abnormalities in the extended SHIRPA screen.

The reversal-learning phase of the set-shifting task yielded an inconclusive result, as no shift cost was observed in wild-type animals for the number of trials to reach criterion. Indeed, the genotype difference that was observed in the number of trials to reach the criterion in the reversal-learning phase was rather caused by lack of shift cost in wild-type animals than a reversal-learning deficit in the *Cntn4*^−/−^ mice. In line with this reasoning, when we compared the outcome of *Cntn4*^−/−^ mice in the reversal-learning phase with the results obtained for C57BL/6J mice obtained in a previous study, we find no genotype difference in this phase of the set-shifting task [6]. In the Barnes maze reversal-learning paradigm, we also did not find a reversal-learning deficit. Based on the summary of these data, we conclude that *Cntn4* disruption has no effect on cognitive flexibility.

Non-ASD-specific effects at adult ages were indicated by increased startle response to auditory stimuli of different high amplitudes and by faster escape hole finding during subsequent days of acquisition in the Barnes maze.

Hyperresponsivity to acoustic stimuli is related to many neurodevelopmental disorders and has also been reported in ASD [22]. In addition, acoustic hyperresponsivity in patients with fragile X syndrome is known to be consistent with animal model data [23]. Similar to our findings, an increased startle response to acoustic stimuli was recently described in children with ASD [24], although these were in response to weak stimuli in contrast to the high amplitudes we found. The observed hyperresponsivity in *Cntn4*-deficient mice was unrelated to anxiety levels, as mice showed similar exploratory behaviors in classical anxiety behavioral tests, such as the elevated plus maze and open field. Although speculative, the increased startle response together with the enhanced acquisition in the Barnes maze could indicate that *Cntn4* deficiency in the C57BL/6J background leads to a state of increased behavioral responsiveness without overt anxiety or avoidance behavior in mice [25].

Together, the findings show that in the C57BL/6J background, disruption of *Cntn4* does not lead to substantial behavioral defects related to autistic development. A limited behavioral penetrance of *CNTN4* mutations on autistic development may be consistent with a recent study that revisited the association of contactins, including *CNTN4*, with ASD [16]. The behavioral phenotype of *Cntn4* mice contrasts other previously studied genetic ASD models [18]. For instance, *Shank3* or *Pten* mice show extensive impairments in social interaction and sensorimotor phenotypes and are therefore regarded as translational models for ASD [26–28].

Although ASD-related phenotypes in *Cntn4*-deficient mice were observed, our findings do have relevance for neurodevelopmental disorder research. The specific phenotypes observed in *Cntn4*-deficient mice may be used to study the mechanisms underlying increased responsiveness or vigilance, a trait observed across many different human disorders such as attention-deficit hyperactivity disorder, post-traumatic stress disorder, and schizophrenia [29–31]. *Cntn4*-deficient mice may serve as a model to study the mechanistic underpinnings of behavioral states in which vigilance is altered. Indeed, common single-nucleotide variants in the *CNTN4* locus have recently been associated with other neuropsychiatric disorders, such as schizophrenia [32], perhaps pointing to a non-disorder specific contribution of this cell adhesion gene in neuropsychiatric pathogenesis. In addition, a role for *Cntn4*, was recently shown in target-specific arborization during development of the accessory optic system [9]. Our study shows the importance of detailed developmental neurological, behavioral, and cognitive characterization of genetic animal models to complement human genetic studies in ASD and related disorders.

## Conclusions

In our test battery, disruption of *Cntn4*, a prominent ASD candidate gene*,* had no effect on cognitive and behavioral development or ASD-specific phenotypes. At adult age, we could detect an effect of *Cntn4* disruption on an adult sensory behavioral and a spatial cognitive feature.

## Additional file

Additional file 1: Detailed information on genotyping, behavioral testing and statistical analyses.

## Abbreviations

ASD: autism spectrum disorder
eSHIRPA: extended SmithKline Beecham, Harwell, Imperial College and Royal London Hospital phenotype assessment
PPI: pre-pulse inhibition

## References

[1] Am. Psychiatr. Assoc.: Diagnostic and statistical manual of mental disorders. 5th ed. Washington, DC; 2013.

[2] Chen JA, Peñagarikano O, Belgard TG, Swarup V, Geschwind DH (2015). The emerging picture of autism spectrum disorder: genetics and pathology. Annu Rev Pathol: Mech Dis.

[3] Pinto D, Delaby E, Merico D, Barbosa M, Merikangas A, Klei L (2014). Convergence of genes and cellular pathways dysregulated in autism spectrum disorders. Am J Hum Genet.

[4] De Rubeis S, He X, Goldberg AP, Poultney CS, Samocha K, Ercument Cicek A (2014). Synaptic, transcriptional and chromatin genes disrupted in autism. Nature.

[5] Abrahams BS, Arking DE, Campbell DB, Mefford HC, Morrow EM, Weiss LA (2013). SFARI Gene 2.0: a community-driven knowledgebase for the autism spectrum disorders (ASDs). Mol Autism.

[6] Molenhuis RT, de Visser L, Bruining H, Kas MJ (2014). Enhancing the value of psychiatric mouse models; differential expression of developmental behavioral and cognitive profiles in four inbred strains of mice. Eur Neuropsychopharmacol.

[7] Zuko A, Kleijer KTE, Oguro-Ando A, Kas MJH, Van Daalen E, Van Der Zwaag B (2013). Contactins in the neurobiology of autism. Eur J Pharmacol.

[8] Kaneko-Goto T, Yoshihara S-I, Miyazaki H, Yoshihara Y (2008). BIG-2 mediates olfactory axon convergence to target glomeruli. Neuron.

[9] Osterhout JA, Stafford BK, Nguyen PL, Yoshihara Y, Huberman AD (2015). Contactin-4 mediates axon-target specificity and functional development of the accessory optic system. Neuron.

[10] Fernandez T, Morgan T, Davis N, Klin A, Morris A, Farhi A (2004). Disruption of contactin 4 (CNTN4) results in developmental delay and other features of 3p deletion syndrome. Am J Hum Genet.

[11] Roohi J, Montagna C, Tegay DH, Palmer LE, DeVincent C, Pomeroy JC (2009). Disruption of contactin 4 in three subjects with autism spectrum disorder. J Med Genet.

[12] Pinto D, Pagnamenta AT, Klei L, Anney R, Merico D, Regan R (2010). Functional impact of global rare copy number variation in autism spectrum disorders. Nature.

[13] Glessner JT, Wang K, Cai G, Korvatska O, Kim CE, Wood S (2009). Autism genome-wide copy number variation reveals ubiquitin and neuronal genes. Nature.

[14] Guo H, Xun G, Peng Y, Xiang X, Xiong Z, Zhang L (2012). Disruption of contactin 4 in two subjects with autism in Chinese population. Gene.

[15] Cottrell CE, Bir N, Varga E, Alvarez CE, Bouyain S, Zernzach R (2011). Contactin 4 as an autism susceptibility locus. Autism Res.

[16] Murdoch JD, Gupta AR, Sanders SJ, Walker MF, Keaney J, Fernandez TV (2015). No evidence for association of autism with rare heterozygous point mutations in contactin-associated protein-like 2 (CNTNAP2), or in other contactin-associated proteins or contactins. PLoS Genet.

[17] Rogers DC, Jones DNC, Nelson PR, Jones CM, Quilter CA, Robinson TL (1999). Use of SHIRPA and discriminant analysis to characterise marked differences in the behavioural phenotype of six inbred mouse strains. Behav Brain Res.

[18] Silverman JL, Yang M, Lord C, Crawley JN (2010). Behavioural phenotyping assays for mouse models of autism. Nat Rev Neurosci.

[19] Bruining H, Matsui A, Oguro-Ando A, Kahn RS, Spijker HM V’t, Akkermans G (2015). Genetic mapping in mice reveals the involvement of Pcdh9 in long-term social and object recognition, and sensorimotor development. Biol Psychiatry.

[20] Pearson BL, Pobbe RLH, Defensor EB, Oasay L, Bolivar VJ, Blanchard DC (2011). Motor and cognitive stereotypies in the BTBR T+tf/J mouse model of autism. Genes Brain Behav.

[21] Seigers R, Loos M, Van Tellingen O, Boogerd W, Smit AB, Schagen SB. Cognitive impact of cytotoxic agents in mice. Psychopharmacology (Berl). 2015;232(1):17–37. 10.1007/s00213-014-3636-9 24894481

[22] Green S, Hernandez L, Tottenham N, Krasileva K, Bookheimer S, Dapretto M: Neurobiology of sensory overresponsivity in youth with autism spectrum disorders. JAMA Psychiatry. 2015;72(8):778-786. 10.1001/jamapsychiatry.2015.0737 PMC4861140 26061819

[23] Chen L, Toth M (2001). Fragile X mice develop sensory hyperreactivity to auditory stimuli. Neuroscience.

[24] Takahashi H, Nakahachi T, Komatsu S, Ogino K, Iida Y, Kamio Y (2014). Hyperreactivity to weak acoustic stimuli and prolonged acoustic startle latency in children with autism spectrum disorders. Mol Autism.

[25] Blanchard DC, Griebel G, Blanchard RJ (2003). Mouse defensive behaviors: pharmacological and behavioral assays for anxiety and panic. Eur J Pharmacol.

[26] Peça J, Feliciano C, Ting JT, Wang W, Wells MF, Venkatraman TN (2011). Shank3 mutant mice display autistic-like behaviours and striatal dysfunction. Nature.

[27] Jiang YH, Ehlers MD (2013). Modeling autism by SHANK gene mutations in mice. Neuron.

[28] Ey E, Leblond CS, Bourgeron T (2011). Behavioral profiles of mouse models for autism spectrum disorders. Autism Res.

[29] Parker JDA, Majeski SA, Collin VT (2004). ADHD symptoms and personality: relationships with the five-factor model. Pers Individ Dif.

[30] Dalgleish T, Moradi AR, Taghavi MR, Neshat-Doost HT, Yule W (2001). An experimental investigation of hypervigilance for threat in children and adolescents with post-traumatic stress disorder. Psychol Med.

[31] Freedman R, Waldo M, Bickford-Wimer P, Nagamoto H (1991). Elementary neuronal dysfunctions in schizophrenia. Schizophr Res.

[32] Schizophrenia Working Group of the Psychiatric Genomics Consortium (2014). Biological insights from 108 schizophrenia-associated genetic loci. Nature.

