# Supplementary material for: Limited impact of Cntn4 mutation on autism-related traits in developing and adult C57BL/6J mice
Source: J Neurodev Disord. 2016 Mar 2;8:6. doi: 10.1186/s11689-016-9140-2 (PMC4782374; doi:10.1186/s11689-016-9140-2)
Supplement: Additional file 1: — Detailed information on genotyping, behavioral testing and statistical analyses. [file 11689_2016_9140_MOESM1_ESM.docx]

# Additional file 1

### Generation and breeding of *Cntn4* knockout mice

Upon arrival in the University Medical Center Utrecht, mice were re-derived, followed by heterozygous breeding for the use in our experiments. The following primers were used for genotyping *Cntn4-*progeny: 5’-tggtagatggatcgatggcaaacatgtccc-3’ (mutant allele forward); 5’-agccccagtttttgcctaagcatt-3’ (wild-type allele forward); 5’-ttcatcactcctgaatcacacatgtcagg-3’ (mutant and wild-type allele reverse). Absence of protein was confirmed by western blot.

### Developmental neurological and behavioral screening

The original SHIRPA primary screen [2] of general health and neurological reflexes was modified and extended with additional tests to increase the number of behavioral domains measured as we have performed previously [3]. Mice were placed in a Perspex jar and recorded for 5 min. The following phenotypes were observed in the jar: body position (active, inactive or excessively active), tremor (present or not), palpebral closure (eyes open or not), coat appearance (well groomed or irregularities like piloerection), whiskers (intact or trimmed), lacrimation (present or not), defecation (count of the fecal boli). Subsequently mice were transferred to an arena made of Perspex (Macrolon type III cage). During the time the mice spent in the arena an automated recording of locomotor activity was made for 5 min using video-tracking software Ethovision (Noldus Information Technology). In the arena the following phenotypes were observed: transfer arousal (freezing or immediate movement), gait (fluid or not), tail elevation (dragging, horizontal or straub tail), startle response (preyer reflex, no response or additional response) and touch escape (response to touch or flight prior to touch while finger approaches). Mice were transferred out of the arena and the following phenotypes were observed: positional passivity (struggling by different types of handling), skin color (color of plantar surface of forelimbs), trunk curl (forward curling with head to abdomen), limb grasping (clasping of rear limbs), pinna reflex (presence of ear retraction), corneal reflex (presence of eye-blink), contact righting reflex, evidence of biting, grip (grasping of grid), vocalization and puberty (presence of sex organs).

Self-grooming was manually quantified based on 5-min video recordings of the time the mice spent in the arena, using video software The Observer (Noldus Information Technology).

As part of the longitudinal screening, all mice were also tested the RotaRod (Ugo-Basile, Varese, Italy) to test the sensorimotor competence [4]. The latency for the time to fall off the rotating beam is a measure for the motor coordination and balance. The beam starts rotating at a speed of 4 rpm. During a 5-min trial the beam is accelerating until 40 rpm. Two passive rotations during two complete turns were also counted as a fall.

### Juvenile social interaction test

Juvenile social interactions were assessed between two genotype matched, non-cage mate animals at postnatal day 21 as was shown previously [5]. Habituation and testing took place in clean polycarbonate type II cages (mice: 22 × 16 × 14 cm) with fresh bedding. Social interactions, digging and grooming were scored manually based on video recordings using The Observer software (Noldus Information Technology).

### Three chamber social approach test

Preference for social approach was measured using the three-chambered apparatus [5]. The apparatus was a rectangular box made of polycarbonate and was divided into three compartments by partitions with openings, which allowed free access to all compartments. The paradigm consisted of three trials each lasting for ten minutes. Initially, mice were habituated to the empty arena for 5 min after which empty wire cages were introduced and the mice were allowed to inspect these cages for another 10 min. The second phase of the test measured social preference involving a 10-min session where the experimental mouse was exposed to an inanimate object, one of the empty wired cages and a wired cage covering a stimulus mouse. The placement of both social and non-social targets was counterbalanced between experimental subjects. Parameters measured were a) time spent in each chamber, b) time spent sniffing each target. Behavioral scoring occurred manually using Observer software (Noldus Information Technology). Both the time the mice spent in the three chambers as well as the time the mice spent sniffing the cages was scored manually using Observer software (Noldus Information Technology).

### **Social recognition test**

Social recognition capacity was tested using out two-day social discrimination paradigm, see our previous studies [2], [4]. Before testing all mice were group-housed. To avoid exposure to odor prior to testing, intruder animals were housed in separate rooms under identical conditions, e.g., non-gender matched. Edding 30 permanent marker (tip 1.5–3 mm, filled with neutral smelling water- based ink, green; Edding International) was used for labeling the intruder animals. Habituation and testing took place in clean polycarbonate type II cages (mice: 22 × 16 × 14 cm) with fresh bedding. Disposable gloves were used for handling the animals and changed after each trial to avoid odor contamination. Test animals were habituated in the test cage for 5 min and initially exposed to an age- and gender-matched A/J conspecific for 2 min and then, after inter-trial intervals (ITI) of 5 min exposed to the familiar conspecific and a first novel A/J conspecific for 2 min. After testing, the test animals were returned to their home cage. On day 2 for the 24 h ITI testing, again a clean cage with fresh bedding was used and the test animal was habituated for 5 min and re-exposed to the same familiar intruder of day 1 and to a different novel intruder animal from a different cage and housing room than the intruder of day 1 for 2 min. Social investigation was defined as the total time mice engaged in social sniffing, anogenital sniffing and allogrooming. These behaviors were manually scored from video recordings using the Observer software (Noldus Information Technology, Wageningen, The Netherlands). The time spent by the test animal in investigating each intruder animal was measured by a trained observer blind to the animal’s genotype.

### Restricted interest and repetitive behavioral patterns

Restricted interest and repetitive patterns of novel object exploration were assessed in the novel object investigation task, during ten-minute exposure to four novel toys, a paradigm described previously [6]. In this test, a blue lego, miniature bowling pin, dice and green marble. Object-exploration behaviors and self-grooming were manually scored from video recordings using the Observer software (Noldus Information Technology, Wageningen, The Netherlands). Analyses of restricted interest and repetitive sequences of object-exploration were performed as reported previously[6].

### Buried food test

Olfactory capabilities were assessed by the latency to locate buried food[7]. Testing took place in bedding-filled transparent plastic cages (42.5 cm x 26.5 cm x 18 cm, length x width x height).

### Set shifting and reversal learning task

Mice were tested on a variant of the reversal/set-shifting task, as we previously reported [2], [4]. Mice were required to learn the location of a hidden food reward in one of two food cups that were placed in a testing cage. Three days prior to this task, mice were housed solitary and food restricted up to 85% of their ad libitum body weight to ensure appropriate motivation. The reward was hidden under a scented digging medium. Odor and digging medium represented two different dimensions, mice had to learn to focus on the relevant dimension and ignore the other dimension during the different learning phases. Different combinations of odors and digging media were used. The task consisted of 8 sub-tasks: simple discrimination, compound discrimination, intra-dimensional shifts I-IV and intra-dimensional shift IV reversal.

During simple discrimination, mice had to associate either a medium or an odor with the reward, the second dimension was kept constant for both cups. During compound discrimination, both dimensions were different, mice had to learn to ignore the non-relevant dimension and focus on the previously learned association between a certain medium or odor and the reward. During the four intra-dimensional shifts, the relevant dimension did not change, but the specific pairings within the dimension were changed in such a way that the animal had to learn a new association in every task. During the reversal task, the cues stayed the same, except that the previously rewarded medium or odor was no longer reinforced. Thus, mice had to switch to choose the previously not rewarded cue. During the extra-dimensional shift, the relevant dimensions were changed (i.e. mice that previously had to associate a medium with the reward now had to associate an odor, and vice versa). Each phase lasted about 8-30 trials, depending on the learning rate. Criterion for acquisition of each sub-task was set at 8 out of 10 correct consecutive trials. The total task duration was 4 days. The latency to find the reward, the number of trials to reach criterion and the number of errors were recorded for each of the individual tasks.

### Barnes maze and reversal learning

The Barnes Maze is a test for spatial learning and memory, performed as previously reported [8]. The test is similar to the Morris Water Maze, with the important difference that this test does not require swimming. As such, the emotional distress of the Barnes Maze is suggested to be lower than the emotional load of the Morris Water Maze. The Barnes maze contains 24 holes situated at the edge of the maze. One of these holes is connected to an escape box. The testing room is equipped with several visual cues mounted on the wall. After multiple training trials, mice learn to locate the escape hole using the visual cues around the maze.

The Barnes maze consisted of a circular grey platform (diameter 120 cm) elevated 100 cm above the floor with 24 holes (4.5 cm diameter) spaced at equal distance 5 cm away from the edge of the platform. One hole was designated as escape hole, and equipped with a cylindrical entrance (4.5 cm diameter x 5 cm depth) mounted underneath the maze providing access to an escape box (15.3 x 6.4 x 6.1 cm) containing a metal stairway for easy access that was not visible unless mice approached the hole closely. Other holes were equipped identical cylindrical entrances, but without escape box. Visual extra-maze cues (50 x 50 cm) composed of black and white patterns were mounted on the walls ~70 cm away from the maze. Three fans surrounding the maze (60 cm away from the maze spaced ~120° apart) produced a variable airflow across the entire maze by a slow 90° horizontal movement, proving both an aversive environment as well as dispersion of any odor cues. Several fluorescent tube lights mounted at the ceiling provided bright illumination (1000 lx). A speaker mounted to the ceiling provided background sound.

Mice received training sessions twice a day, in the morning and afternoon. Mice were introduced in an opaque cylinder placed in the center of the maze, after which the experimenter left the room and closed the door. The cylinder was pulled upwards 30 s later, and mice could explore the maze to locate the escape hole. If the latency to enter the escape hole exceeded 300 s, mice were gently guided toward the escape hole. During the first 2 habituation sessions, the escape box contained cage enrichment of a mouse's own home cage, and once in the cylinder, mice were left in there for 60 s. After each mouse, the platform and escape box were thoroughly cleaned with 70 % ethanol. The platform was rotated 90° after each trial to avoid the use of any odors cue. During a 300 s probe trial the escape hole was identical to all 23 other holes.

The path travelled by a mouse was video tracked by an overhead camera and analyzed using Viewer 2 software with Barnes maze plugin (BIOBSERVE GmbH, Bonn, Germany). The distance and latency to reach the target location were recorded. Barnes maze training consisted of two training sessions per day, for five days. On the eighth day, two additional training sessions were given. Reversal learning was forced by replacing the escape to the other side of the board on day nine and ten.

### Acoustic startle and pre-pulse inhibition

Acoustic startle and PPI were measured during one 45-min session in four Plexiglas cylinders in ventilated sound-attenuating chambers (Med Associates, St. Albans, VT), placed on separate heavy passive vibration-free tables (Newport Corporation, Irvine, CA) as reported before [4]. During testing, a separate speaker provided white noise background of 65 dB. The intensity of the startle stimuli was calibrated with a microphone placed inside the Plexiglas cylinders in a closed chamber, while white background noise was switched off. Hence, the reported prepulse and pulse intensities are lower than the actual cumulative sound pressure level during testing (e.g., a 65 dB pulse in addition to a 65 dB background noise add up to 68 dB intensity). The session started with a habituation period of 5 min, followed by a total of 260 trials with pseudo-randomized interval periods (5-15 s) consisting of acoustic startle trials with white noise bursts at various intensities (65, 70, 75, 80, 85, 90, 95, 100, 105, 110, and 115 dB; 10 trials per intensity) and prepulse inhibition trials with white noise bursts at various prepulse intensities (0, 65, 67 and 71 dB; 30 trials per prepulse intensity; startle intensity always 120 dB) in pseudo randomized order such that all 4 boxes produced startle stimuli at exactly the same time. In half of the prepulse trials, onset of white noise prepulse stimuli (20 ms; 1 ms programmed rise/fall time) and startle stimuli (40 ms; no programmed rise/fall time) was separated by a 30-ms, in the other half by 100-ms interval. In each trial, the highest startle intensity peak (in relative machine units) was collected during the 100-ms interval after the startle stimulus, from which the individual mean highest startle intensity peak during the 100-ms null-period prior to prepulse stimuli was subtracted. The ISI of 30 ms was used as the PPI in mice is at its maximum around 30-ms ISI [9] whereas for humans the maximum PPI is found at 100 ms [10]. The equipment was calibrated to allow for a wide range of startle intensities, however, the force generated by some mice at the highest pulse intensities could exceed the dynamic range of the equipment (maximum of 2047 units) artificially reducing the percentage of PPI in subsequent analyses. Therefore, when the number of such censored 120-dB pulse trials was more than 33% (e.g., more than 5 out of 15) no PPI was calculated. The percentage of PPI was calculated as follows: PPI = 100 * [(mean startle intensity pulse) - (mean startle intensity pulse with prepulse) / (mean startle intensity pulse)].

### Anxiety-like behavior

As we reported previously, anxiety behavior was measured in an open field test and in the elevated plus maze test, making use of the natural tendency of rodents to avoid open spaces in a novel environment [3]. Mice were exposed to an elevated maze that was cross-shaped and consisted of a central platform and four arms, two of which are enclosed. Mice were put in on the center platform, facing an open arm, and allowed to freely explore the maze for 5 min. The time spent on, and number of entries into the open and closed arms as well as the total distance travelled were recorded using an overhead camera and video tracking software (EthoVision 7.0, Noldus Information Technology, Wageningen, The Netherlands).

The open field consisted of a circular arena with a diameter of 80 cm. Mice were exposed once for a 15 min session. Mice were placed near the wall of the arena. After each trial, the apparatus was thoroughly cleaned using paper towels and Trigene solution (0.5%). An overhead camera connected to a PC with video tracking software (EthoVision 7.0, Noldus Information Technology, Wageningen, The Netherlands) recorded the movement of each animal to measure the total amount of time the mouse spent in the arena center.

### Statistical analyses

Genotype differences in each single trial were determined and analyzed using one-way ANOVA (owANOVA) [3]. Social approach in the 3-chamber social interaction task was additionally tested using a paired t-test, comparing the time investigating the object versus investigating the mouse. Social discrimination ratios in the social recognition paradigm were additionally tested against chance level (a ratio of 0.5) within strain for each interval (T1 and T24) using a one-sample T-test [3]. For repeated measurements, a repeated measures ANOVA (rmANOVA) was performed with ‘time’ as within-subjects factor and ‘genotype’ as between-subjects factor [3]. In case of a significant F-value, post-hoc comparisons were performed using Dunnet’s test with wild type as control group. Genotype differences in startle response were compared using the MANOVA Pillai’s trace statistic as the Mauchly’s indicated violation of sphericity. PPI data were compared using a 2-wayANOVA. Non-normally distributed SHIRPA scores were compared using a non-parametrical Kruskal–Wallis test with Mann–Whitney U tests for post-hoc comparisons. Significance was set at p<0.05. SPSS 20.0 for Windows was used for analyses

## References

1. Kaneko-Goto T, Yoshihara S-I, Miyazaki H, Yoshihara Y: **BIG-2 mediates olfactory axon convergence to target glomeruli.** *Neuron* 2008, **57**:834–46.

2. Rogers DC, Jones DNC, Nelson PR, Jones CM, Quilter CA, Robinson TL, *et al*.: **Use of SHIRPA and discriminant analysis to characterise marked differences in the behavioural phenotype of six inbred mouse strains**. *Behav Brain Res* 1999, **105**:207–217.

3. Molenhuis RT, de Visser L, Bruining H, Kas MJ: **Enhancing the value of psychiatric mouse models; differential expression of developmental behavioral and cognitive profiles in four inbred strains of mice.** *Eur Neuropsychopharmacol* 2014:1–10.

4. Bruining H, Matsui A, Oguro-Ando A, Kahn RS, van ‘t Spijker HM, Akkermans G, *et al*.: **Genetic mapping in mice reveals the involvement of Pcdh9 in long-term social and object recognition, and sensorimotor development**. *Biol Psychiatry* 2015:1–11.

5. Silverman JL, Yang M, Lord C, Crawley JN: **Behavioural phenotyping assays for mouse models of autism.** *Nat Rev Neurosci* 2010, **11**:490–502.

6. Pearson BL, Pobbe RLH, Defensor EB, Oasay L, Bolivar VJ, Blanchard DC, *et al*.: **Motor and cognitive stereotypies in the BTBR T+tf/J mouse model of autism.** *Genes Brain Behav* 2011, **10**:228–35.

7. Yang M, Crawley J: **Simple Behavioral Assessment of Mouse Olfaction**. *Curr Protoc Neurosci* 2009.

8. Seigers R, Loos M, Van Tellingen O, Boogerd W, Smit AB, Schagen SB: **Cognitive impact of cytotoxic agents in mice**. *Psychopharmacology (Berl)* 2015:17–37.

9. Yeomans JS, Bosch D, Alves N, Daros A, Ure RJ, Schmid S: **GABA receptors and prepulse inhibition of acoustic startle in mice and rats**. *Eur J Neurosci* 2010, **31**:2053–2061.

10. Braff D, Grillon C, Geyer M: **Gating and habituation of the startle reflex in schizophrenic patients.** *Arch Gen Psych* 1992:206–215.
